# Macao Guangdong music through the lens of ethnography of musical performance

**DOI:** 10.3389/fpsyg.2026.1856681

**Published:** 2026-07-17

**Authors:** FanLe Kong, Zhong Zheng, Chang Xu, YueRu Zhang

**Affiliations:** 1 Faculty of Humanities and Arts, Macau University of Science and Technology, Macao SAR, China; 2 The Institute of Chinese Intangible Cultural Heritage, Sun Yat-sen University, Guangzhou, China; 3 Macao Chinese Orchestra, Macao, Macao SAR, China

**Keywords:** cross-cultural identity, embodied practice, ethnography of musical performance, Guangdong music, Macao

## Abstract

This study adopts the ethnography of musical performance as its methodological framework. Taking the Macao Guangdong Music Friendship Association as its primary field site, it employs a three-tiered “body–event–context” model to examine the living practices of a traditional music society in an urban setting. Through participant observation of rehearsals, teaching sessions, community festivals, and international performances, the research focuses on a micro-ethnographic thick description of the musicians' embodied practices, including playing postures, aural habits, and non-verbal interactions. The findings reveal that musical style is shaped through “embodied practice” and immersion in the local soundscape, giving rise to a distinctive “Macao taste” (Macao wei). The music society performs strategic cultural identity work across different social arenas through repertoire selection and embodied narratives. Furthermore, the stratified repertoire and original works such as *Shengshi Haojiang* (*Prosperous Macao*) constitute a living sound archive that documents and participates in local cultural-historical narratives and identity construction.

## 1 Introduction

As a historical city where Chinese and Western civilizations have intersected for over four centuries, Macao possesses a rich and distinctive cultural soundscape. However, compared to the extensive scholarly attention paid to its tangible cultural heritage and syncretic folk customs, the practice of Guangdong music, which is deeply rooted in the daily auditory experiences and social lives of the local Chinese community, has long remained at the periphery of academic research, constituting a notable research gap. Existing studies have predominantly focused on the historical evolution and canonization of Guangdong music within mainland China (Li and Huang, 2003; Wan, 2010). According to the Guangzhou City Chronicle, the transmission system for Guangdong music activities in the Guangzhou area had already become relatively well-established by the late Qing and early Republican periods (Guangzhou Local Chronicles Compilation Committee, 2000). In contrast, the living practices and contemporary transmission of Guangdong music in Macao's unique cross-cultural context have received insufficient attention.

Guangdong music originated in the Pearl River Delta and matured during the rapid urbanization and commercialization of the 20th century. From the “bayin ban” (eight-man ensemble) of rural society to the “jingshen yinyue” (light music) of urban teahouses and the “shidai qu” (popular tunes) of the recording industry, its developmental history is itself a narrative of fluidity, adaptability, and creativity (Li and Huang, 2003; Huang, 2006). This paper focuses on the Macao Guangdong Music Friendship Association, an amateur music society that inherits the Lingnan “sihuoju” (private music club) tradition of socializing through music while simultaneously developing new dimensions within Macao's distinctive socio-cultural environment. Established in 2012, the association's members are primarily middle-aged and elderly enthusiasts. They actively participate in Macao's cultural life through regular rehearsals, community performances, international cultural exchanges, and educational outreach in schools.

Faced with such an urban music society situated in a complex cross-cultural context, traditional musicological approaches—which focus primarily on scores, repertoire, and historical lineages—cannot adequately capture the tacit dimensions of musical knowledge transmission, the social interaction mechanisms inherent in performance, or the cultural strategies and identity politics underlying practice. Therefore, this study adopts the ethnography of musical performance as its core methodology. Originating from the “performance turn” in anthropology and folklore, this orientation treats music as an emergent, situated social behavior and communicative event rather than as a static text. As Bauman (1977) argued in Verbal Art as Performance, performance is characterized by “emergent” qualities and situatedness. Stokes (1994), in Ethnicity, Identity and Music, emphasized that musical performance constitutes a crucial arena for the negotiation of cultural identity. Within the Chinese ethnomusicological context, Xiao and Li (2019), in “Theory and Practice of the Ethnography of Musical Performance,” systematically introduced this approach, advocating for deep participant observation to document and interpret the entire process of musical performance, with particular attention to performers' embodied practices, the emergent creativity of performance events, and the dynamic interplay between performance and its socio-cultural context.

Based on this theoretical perspective, this paper addresses two interrelated core questions: first, how is the stylistic essence of Guangdong music transmitted in Macao through “embodied practice,” and how does prolonged immersion in the local soundscape shape a distinctive local aesthetic perception—referred to in this paper as the “Macao taste” (Macao wei)? Second, how do the layered structure of the association's repertoire and original compositions such as *Prosperous Macao* constitute what this paper terms a? “living sound archive” that documents and participates in local cultural-historical narratives and identity construction? At the micro-level, this paper focuses on the musicians' performance postures, auditory habits, and non-verbal interactions to reveal the internal mechanisms of skill transmission and the formation of bodily habitus. At the macro-level, it situates these concrete practices within Macao's historical strata and cultural context to examine how music becomes an agentive medium for documenting and participating in local narratives.

This study aims both to fill a gap in the case study research on Macao Guangdong music and to systematically apply and reflect upon the methodological approach of the ethnography of musical performance through this case. Fieldwork was conducted from January to December 2025. The researcher, acting as a “performer-participant,” attended the association's regular rehearsals, which were held twice weekly (Mondays and Fridays, 9:00–11:30 a.m.), each lasting approximately 2.5 h, at the Macau Street General Community venue. In addition, the researcher observed two representative live performances in 2025: (1) “Celebration of the 80th Anniversary of the Victory of the Chinese People's War of Resistance Against Japan” (July 13, 2025, at Escola de Cidadania de Macau Auditorium); (2) “Concert in Celebration of the 26th Anniversary of Macao's Return and Ode to the Lotus Guangdong Music” (December 14, 2025, at Tai Wo Industrial Building, 10th floor). To supplement the limited number of live observations, the researcher also reviewed archival concert videos provided by the association, focusing on performing postures, ensemble coordination, and non-verbal interactions across different occasions.

The researcher conducted semi-structured in-depth interviews with 12 members of the association, including the president, the chairman, and senior instrumentalists. The majority of the interviewees were elderly, most around 70 years of age. Each interview lasted between 45 and 90 min, was audio-recorded, and transcribed verbatim. The interview data were analyzed using thematic analysis (Braun and Clarke, 2006): first, open coding was performed on the transcripts to extract initial concepts related to “embodied practice,” “strategic identity performance,” and “local narrative”; then, themes were clustered and refined; finally, the themes were theoretically aligned with the “Body–Event–Context” framework of musical performance ethnography. Field notes, interview data, and video analysis were cross-validated to achieve triangulation.

## 2 Body·event·context: an analytical framework

To systematically address the research questions outlined above, this study integrates performance theory, embodied practice, and contextual analysis to construct a three-tiered analytical framework of “Body–Event–Context.” This framework aims to overcome the limitations of any single theoretical tool and to capture the emergent processes through which Macao Guangdong musical culture is created, performed, and transmitted in concrete situations.

From the perspective of performance theory, Richard Bauman defines performance as “a special, artistic mode of communication” characterized by “emergent quality” and situatedness (Bauman, 1977). When this perspective is introduced into ethnomusicology, the analytical focus shifts from the static “musical work” to the dynamic “musical performance event,” attending to how music is creatively produced and endowed with meaning on the spot, through interactions between performers and participants in specific time–space settings. Consequently, the study of the Macao Guangdong Music Friendship Association must immerse itself in the socio-cultural processes embedded in every rehearsal, performance, teaching session, and other events.

The transmission of social memory relies heavily on what Paul Connerton calls “embodied practice”—the transmission of knowledge through repeated bodily habits, movements, and postures (Connerton, 1989). In the transmission of Guangdong music, the tacit knowledge that constitutes the core of its style—such as yunwei (nuanced flavor), qikou (breathing points), and xinban (internal pulse)—is precisely conveyed through hands-on demonstration, imitation, and bodily discipline between master and apprentice. The body thus becomes the primary interface through which this study observes cultural transmission, aesthetic perception, and creative fusion. This perspective resonates with the “carnal musicology” approach in music scholarship, emphasizing that the performer's body is not merely a vehicle for technique but also a site of meaning production (Le Guin, 2006). At the same time, musical performance is a crucial arena for the public negotiation and reconstruction of cultural identity (Stokes, 1994). In Macao's cross-cultural context, the ensemble's repertoire selection, staging behavior, and creative practices must all be interpreted within their specific historical strata and social milieu.

In summary, performance theory directs the analytical focus to the “event,” embodied practice theory delves into the “body,” and contextual analysis requires us to always situate “events” and “bodies” within the historical and social networks that embed them. Together, the three constitute a mutually supporting analytical whole. Based on the above theories, this study unfolds its analysis across three interrelated levels. At the micro-level, it focuses on bodily interactions in rehearsals and teaching, analyzing the formation of technical transmission, bodily habitus, and local auditory aesthetics (the “Macao taste”). At the meso-level, it provides thick descriptions of different types of performance events—community festivals, international stages, educational outreach—to interpret how the ensemble strategically performs cultural identity through performance frames and repertoire choices. At the macro-level, it situates micro- and meso-level practices within Macao's historical and cultural strata, analyzing how the layered structure of the repertoire and original works such as *Prosperous Macao* participate in local narratives and identity construction.

The ethnographic grounding of this framework derives from the author's participant observation of the Macao Guangdong Music Friendship Association in 2025.

## 3 Micro-analysis: embodied practice, habitus formation, and the shaping of local auditory perception

### 3.1 Embodied transmission and the transfer of tacit knowledge: “discipline” and “metaphor” in the rehearsal hall

In the musical practice system of the association, musical notation (whether gongchepu or numbered notation) primarily plays the role of a “skeletal score” or “framework prompt.” The “nuanced flavor” that endows the music with vitality and stylistic identity—such as the precise timing and dynamic layering of ornamental jiahua (embellishments), the delicate portamento in melodic lines, and the breathing points (qikou) marking phrase beginnings and endings—is transmitted almost entirely through a body-mediated mechanism of demonstration, imitation, and interaction.

Among these, “hands-on” bodily discipline is the most direct and efficient teaching method. In the field, I repeatedly observed that when senior musicians instructed novices on adjusting the bowing technique of the gaohu (a high-pitched two-stringed fiddle), they rarely offered abstract theoretical explanations of mechanics or aesthetics. Instead, they approached closely, gently placing a hand on the student's right forearm to guide it through the complete curved trajectory from the frog to the tip of the bow. Accompanied by verbal guidance such as “Feel the bow hair ‘biting' the string, being smoothly ‘sent' forward, not harshly ‘pressed' down,” the correct pattern of force transmission, movement speed, and spatial trajectory was directly “inscribed” into the apprentice's bodily schemas (i.e., the internalized representations of actions, postures, and sensations). This process is a typical manifestation of what sociologist Paul Connerton termed “embodied practice”: the memory and skills of a society (or a specific musical community) are transmitted and perpetuated through the repeated performance of bodily actions (Connerton, 1989).

When direct physical adjustment cannot fully convey subtle and abstract musical requirements, the musicians skillfully employ a series of vivid metaphors rooted in local life experience, thereby constructing a cognitive bridge for communicating bodily sensations. For example, when demanding a “round” tone, the master might say, “It should feel like gently stroking a smooth egg.” When criticizing a rigid, lifeless performance, they might say, “Like a piece of wood without breath.” The value of these metaphors lies not only in rhetoric but also in their ability to effectively guide the apprentice to translate familiar, everyday bodily feelings (such as touch and kinesthesia) into the “body intentionality” and motor programs required for specific performance actions.

The process of mastering style is thus a history of forming a bodily habitus. A yangqin (hammered dulcimer) player recalled his experience learning jiahua: “At the beginning, I rigidly memorized the master's fixed notation; it was very stiff. Later, after listening more and playing more, one day my hands knew how to go on their own. The embellishments flowed out naturally, without my mind having to think.” The phrase “my hands know how to go” vividly marks the internalization of specific musical syntax and body movement patterns, which become an automated “second nature” or “sense of practice” no longer requiring conscious control.

### 3.2 Bodily coordination in ensemble playing and the forging of a “social body”: breathing, micro-interactions, and “internal pulse”

When musical practice moves from individual practice to collective ensemble, the rehearsal hall transforms from a space for transmitting individual skills into a crucible for forging a “social body” and collective musical perception. The ensemble style of Guangdong music's sihuoju (private music club) does not rely on written scores or an authoritative conductor. Its essence lies in “mutual understanding through the heart,” and the achievement of such a high degree of tacit coordination depends on a sophisticated system of bodily coordination and shared sensation.

The beginning of an ensemble is often unified by a non-verbal yet crucial bodily signal: breathing. Usually, the gaohu player acting as the “head frame” (lead performer) takes a slight but deep inhalation before the start of a musical phrase, accompanied by a subtle lifting of the shoulders. This bodily cue is instantly captured by the other musicians, who almost simultaneously adjust their breathing and enter a state of readiness. This ritual act of “breathing in together” not only synchronizes the timing of the attack at the physiological level but also instantly constructs a temporary, highly focused “musical community” at the psychological and perceptual levels, laying a mind–body foundation for subsequent coordinated interpretation.

During the performance, the balance, dialogue, and development among the parts are maintained by an improvised, highly contextualized system of bodily “micro-interactions.” The “head frame” uses subtle changes in facial expression, slight nods or turns of the head, to indicate subtle tempo shifts or the entry of a particular part. Between wind and bowed-string players, quick eye contact often confirms the phrasing and shared breathing points. When the performance reaches a highly engaged and tacitly coordinated passage, the musicians' upper bodies often unconsciously move in synchronized, tiny oscillations. This phenomenon of “synchrony” at the bodily level is not only an external manifestation of a highly unified internal musical pulse but also serves as positive feedback, continuously reinforcing the emotional cohesion and collective excitement among the performers.

The “internal pulse” (xinban) is a core concept for understanding this collective bodily wisdom. It differs from the mechanical, evenly divided time of a metronome; rather, it is a collective rhythmic pulse full of elasticity, breath, and internal tension. One seasoned musician explained: “We don't listen to the rigid beat; we listen to the entire musical ‘flow.' It's like a team paddling a dragon boat—you don't just stare at your own paddle; you feel the speed and combined force of the whole boat, and then your hands naturally follow that energy.” Temporarily “suspending” one's own internal sense of rhythm to keenly perceive and merge into the collective rhythmic flow co-created and maintained by all participants is the highest level of embodied practice. It marks the successful transformation of an individual's bodily habitus into a deep perception of, and belonging to, the rhythm of the “social body.” This collective bodily synchrony and rhythmic perception is referred to in music research as “entrainment” (Clayton et al., 2005), which illuminates the neural and behavioral mechanisms of intercorporeal coordination in musical performance.

### 3.3 Immersion in the local soundscape and the formation of the “Macao taste” auditory aesthetic

The emergence of the “Macao taste” (Macao wei) stems not only from conscious skill transmission and rehearsal honing but is also deeply rooted in the unconscious shifts in auditory habits and aesthetic preferences resulting from the musicians' long-term immersion in Macao's unique urban soundscape. The body is the interface through which the environment is perceived; prolonged exposure to a specific sonic environment reshapes the body's perceptual structure and aesthetic orientation, which in turn permeates bodily expression and tone quality during performance (Ingold, 2000, p. 185–192).

Several musicians with extensive performance experience in Guangdong, Hong Kong, and Macao have pointed out, quite consistently, that when the association performs certain traditional pieces, the overall tempo tends to be “a little slower” and “a little looser” than interpretations in the core Cantonese region. One gaohu player originally from Guangzhou admitted: “When I first started playing with them, I always felt a little ‘out of sync,' as if I was perpetually half a beat behind. But after living in Macao for a long time, I realized that this ‘slowness' is actually quite relaxed and free of excessive ambition.” This “slowness” and “looseness” are difficult to attribute to any individual's deliberate design; they appear more as a collective, unconscious bodily rhythmic preference. It may be implicitly co-generated with Macao's relatively unhurried pace of life and the city's temperament of “calmness” and “tolerance” forged through the long-term coexistence, encounter, and fusion of Chinese and Western cultures (Dai, 2020). This territorial “sense of time” is absorbed through the body and then expressed as a regional feature in musical tempo and rhythmic treatment.

Macao's daily auditory environment is highly heterogeneous and hybrid: the soft sounds of Cantonese opera teahouses, the resonant bells of Catholic churches, the lively rhythms of Portuguese folk dance, the electronic beats of contemporary pop music, and the bustling sounds of urban entertainment districts all interweave to form a unique “urban soundscape.” As Dai (2020, p. 39) observes, in Macao, “whether in Chinese music or Western music, religious or secular, folk or official, each category has its own developmental threads and contemporary achievements.” This long-term, high-intensity cross-cultural auditory exposure may have subtly broadened the local musicians' tolerance and aesthetic inclusiveness for timbral mixing. When discussing the introduction of cello or other low-pitched instruments to enrich the texture, or when facing Western harmonic backgrounds in the performance of *Prosperous Macao*, the musicians generally display a flexible attitude of experimentation, adaptation, and seeking integration. One veteran musician's comment is representative: “Macao is a small place; you hear all kinds of sounds. The sound of the gaohu and the sound of a church organ can actually be very harmonious. The key is to find a way to ‘fuse' them, not to let them fight.” The conscious pursuit of “fusion” and a preference for a timbral aesthetic that allows a little “complexity” within clarity and seeks overall “harmony” amidst individual expression may be the concrete manifestation of the “Macao taste” in the dimension of timbral aesthetics.

The most vivid example occurs in the creative moments of improvised jiahua (embellishment). After a particularly spirited improvisational solo, a zhongruan (mid-range plucked lute) player expressed some surprise: “I don't know why, but I just accidentally played a little bit of a sighing, circling flavor reminiscent of Portuguese fado.” He could not clearly identify the direct source of this musical fragment, but it may well be a “trace” or “fragment” left in long-term auditory memory by Macao's diverse soundscape. At the moment of fully engaged creative expression, it was unconsciously invoked and blended into the melodic ornamentation logic of traditional Guangdong music. These sporadic, individualized auditory “dialects” or “vocabularies,” while not yet constituting a systematic new stylistic grammar, clearly indicate that local auditory experience has begun to infiltrate and subtly alter the deep texture of musical expression.

### 3.4 Conclusion: the bodily as the foundation of cultural practice

This section, through detailed micro-ethnographic thick description, puts into practice the “body/embodied practice” dimension of the analytical framework. The analysis shows that the core mechanism of the “living” transmission of Guangdong music in Macao lies in embodied practice: stylistic elements are inscribed as the musicians' bodily habitus through “hands-on” discipline, guidance by local metaphors, and repeated physical drilling. The essence of ensemble playing lies in the coordinated forging of a “social body”—the synchronization of breathing, bodily micro-interactions, and collective perception of the “internal pulse.” The so-called “Macao taste” is first and foremost a “sense of body”—a more relaxed rhythmic inertia, a more inclusive concept of timbre, and a reservoir of unconsciously mixed melodic vocabulary waiting to be called upon, all nurtured in the local sonic ecology. The body is not merely a passive container of tradition but an active “interface” that perceives, filters, absorbs, and transforms local cultural experience.

## 4 Meso-level events: strategic identity performances in performance contexts^1^

This section employs ethnographic thick description to examine how the Macao Guangdong Music Friendship Association strategically adjusts its “performance frames” (Goffman, 1959) in different performance contexts, thereby performing multiple cultural identities. For analytical clarity, three role concepts used in this section are defined as follows. “Culture guardian” refers to the association's role in community festivals, where it performs classic repertoire with reserved demeanor, thereby maintaining traditional cultural boundaries and reinforcing internal identity. “Cultural bridge” refers to its role in educational outreach, where it deconstructs knowledge and offers hands-on interaction to connect tradition with potential new followers. “Cultural ambassador” refers to its role on transcultural stages and in thematic celebrations, where it employs hybrid narratives and symbolic fusion to represent Macao in external cultural display and dialogue. As Turino (2008) argues, musical performance serves multiple functions in identity construction. Christopher Small further extends this perspective with his concept of “musicking,” emphasizing that the meaning of music lies not in the work itself but in the interpersonal interactions and meaning-making that occur during performance as a social act (Small, 1998). The present case shows that the association's cultural practice is not a single model but a repertoire of strategic performance frames, whose roles are flexibly switched among the above three according to the definition of the situation (Turino, 2008; Small, 1998).

### 4.1 “Culture guardian” in community festivals: ritual performance and the consolidation of internal identity

In traditional folk belief festivals such as the Mazu Birthday and Nezha Birthday in 2018, the association performs in temple theaters or festival squares. The audience consists mainly of local long-term residents and elderly devotees. The performance frame is highly traditional and inward-oriented, and the core strategy is to construct and perform the role of “culture guardian.” The repertoire is strictly limited to Guangdong music classics such as Dragon Boat Race (Sai Long Duojin), Joy and Peace (Yule Shengping), and Rain Pattering on Banana Leaves (Yu Da Bajiao). By performing these “canons,” the association confirms the community's cultural roots and historical continuity on an auditory level. The musicians dress in unified formal attire, their bearing is solemn, and during performance they focus their gaze on the instruments or scores, deliberately downplaying direct entertainment-oriented interaction with the audience. This creates an introverted, almost ritualistic atmosphere of focused attention. As one senior member put it: “Performing in front of the temple feels completely different. It is as if we are not simply playing for people, but together with the festival and this place, we are ‘practicing' a tradition.” The primary function of performance here is to “honor the gods” and to “entertain the people” (the internal “we”), aiming to maintain the community's cultural boundaries and reinforce internal identity and a sense of historical continuity (Turino, 2008).

### 4.2 “Cultural bridge” in educational outreach: enlightenment, accessibility, and intergenerational transmission

In settings such as primary and secondary schools during 2025 (where the audience consisted of students and young parents who might be unfamiliar with Guangdong music), where the audience consists of students and young parents who may be unfamiliar with Guangdong music, the core goal of the performance shifts to “cultural enlightenment” and “intergenerational transmission.” The association transforms into a “cultural bridge” connecting tradition with the general public. The repertoire is short, lively, and melodically bright, sometimes even including short pieces adapted from popular tunes. The format often adopts a “talk + demonstration” approach: the host explains and the ensemble demonstrates, deconstructing the aesthetic experience into knowledge about instruments, technical highlights, and cultural stories, and then rebuilding a ladder of understanding for the audience. The climax typically involves inviting the audience, especially children, to engage in simple physical interactions, such as touching an instrument or trying to produce a sound. This brief embodied experience is crucial—it turns tradition from a distant “other” to be watched into a tangible “my own experience.” As one musician who has participated in many school events remarked, “The sparkle in the children's eyes when they produce a sound with their own hands speaks more deeply than any theoretical explanation.”

### 4.3 “Cultural ambassador” on transcultural stages and in thematic celebrations: image building and communal cohesion

In international cultural exchanges (e.g., performances in Japan, South Korea, and Thailand in 2014), regional festivals (such as the “Greater Bay Area Guangdong Music Gala”), and thematic concerts marking major historical anniversaries (e.g., July 13, 2025 for the 80th anniversary of the Victory of the Chinese People's War of Resistance Against Japan, and December 14, 2025 for the 26th anniversary of Macao's return) , the audience expands to include cultural officials, international tourists, fellow artists, and a broader, diverse public. The function of the performance shifts from “internal cohesion” to “external display” and “cultural dialogue.” The association plays the role of “cultural ambassador,” with the dual character of both “Macao representative” and “Greater Bay Area member.” In celebratory scenarios, it further rises to the role of “ceremonial performer,” using grand narratives to consolidate communal emotions.

The program is a carefully crafted “cultural narrative.” Classic pieces are often presented in “highlight versions” or newly arranged forms to display the essence of the tradition, while original works emblematic of Macao's identity, such as *Prosperous Macao* (Shengshi Haojiang) and Ode to the Lotus (Lianhua Song), are placed in prominent positions (e.g., the finale), creating a linear narrative structure of “tradition–fusion–innovation.” Visual symbols are adjusted accordingly: costumes that blend Chinese and Western elements, stage designs incorporating motifs such as the lotus flower—all help construct an easily recognizable and distinctive cultural image. Performances are often preceded by program notes or introductions, and the performers' body language becomes more expressive and communicative. In a media interview before the “Concert in Celebration of the 26th Anniversary of Macao's Return” on December 14, 2025, the association's president explained: “We hope that our music, and even our attire, can tell the outside world that we come from a special place—Macao.” In thematic celebrations, the scale of the performance is larger, sometimes involving collaborations with other ensembles, creating a majestic “grand gala” atmosphere. The stage presentation is magnificent and solemn, aiming to create a dignified yet festive ritual ambience. At such moments, the performance transcends everyday transmission and becomes a public ritual, reinforcing community members' sense of identity and pride in their shared history, achievements, and future through collective listening and emotional resonance.

In sum, the association performs the “culture guardian” in community festivals by adhering to classics and displaying introverted solemnity; it performs the “cultural bridge” in educational settings through deconstruction, interaction, and bodily initiation; and it performs the “cultural ambassador” on transcultural stages and in thematic celebrations through hybrid narratives and symbolic fusion. These frames are flexibly deployed according to the definition of the situation, fully demonstrating the “performative,” “processual,” and “strategic” nature of cultural identity (Turino, 2008). The association is not a passive vessel of tradition but an active socio-cultural agent. It knows well how to adjust its musical expression, bodily presentation, and symbolic deployment in different fields of power and audience expectations, thereby effectively negotiating, positioning, and proclaiming its multiple cultural identities, and keeping Guangdong music—as a tradition—agentive and relevant in the contemporary cultural-political ecology of Macao.

## 5 Macro-level picture: repertoire stratification, creative agency, and sonic local narratives

Having examined the micro-level of bodily perception and the meso-level of strategic performance events, the analysis of musical performance ethnography must ultimately situate its findings within the historical and cultural macro-context that shapes and endows them with meaning. This section places the musical practices of the Macao Guangdong Music Friendship Association back into the layered field where local, regional, and global forces intersect. By analyzing the “historical stratification” of its repertoire and the “creative agency” embodied in original works such as *Prosperous Macao* (Shengshi Haojiang), the section explores how music not only reflects social action but also, as an agentive “sonic text,” participates in the imaginative construction of local historical narratives, cultural representation, and identity.

### 5.1 A living sound archive: historical stratification of the repertoire and sonic inscription of cultural dialogue

The repertoire that the association rehearses and performs in daily practice is far from a random collection; rather, it is a sonic repository with a clearly layered historical structure—what this paper terms a “living sound archive.” This archive can be broadly divided into three mutually overlapping and dialogical strata. Each stratum corresponds to a key moment of cultural adaptation in the development of Guangdong music and also reflects Macao's unique situation within the modern and contemporary historical transformations of China and the world.

1. Foundational stratum: the “Auditory Mother Tongue” of Lingnan and Stylistic Grounding. This stratum consists mainly of classic pieces from the “golden age” of Guangdong music in the 1920s−1940s and earlier, such as Rain Pattering on Banana Leaves (Yu Da Bajiao), Autumn Moon over the Calm Lake (Pinghu Qiuyue), and Dragon Boat Race (Sai Long Duojin). These works established the basic stylistic paradigm of Guangdong music as a mature urban genre: flowing, elegant melodic lines; lively, agile rhythmic patterns; ornamental grammar of jiahua (embellishment) and portamento; and the characteristic timbral combinations represented by the “five-piece ensemble” (wujiatou). For the association, masterful performance of these pieces confirms its artistic lineage and maintains the Cantonese cultural root (“Guangfu-ness”), providing its core aesthetic coordinates, technical norms, and emotional expressive vocabulary.
2. Influenced stratum: imprints of professionalization and nationalization. This stratum mainly comprises works created between the 1950s and the 1980s under the influence of mainland China's socio-cultural and compositional contexts, such as Trying the Horse in Spring Suburbs (Chunjiao Shima) and Early Spring in Mountain Villages (Shanxiang Chunzao). While retaining a distinct Guangdong musical flavor in their melodic contours, these works exhibit more regularized formal structures, reflecting the impact of professional compositional practices on the traditional genre. Their inclusion and preservation in the association's active repertoire reflect, on the one hand, the continuing cultural ties between Macao and the mainland (especially Guangdong), and on the other, the “fluid” character of Guangdong music as an open tradition that actively absorbed and integrated new musical languages and the spirit of the times during a specific historical period.
3. Self-conscious stratum: seeking, constructing, and voicing “Macao Sound.” This is the most contemporary and locally self-conscious stratum. It is represented by works created, commissioned, or arranged by the association that focus on Macao's local themes, history, and customs, with the large-scale Guangdong music symphonic poem *Prosperous Macao* as its quintessential example. The emergence of this stratum marks a shift in the association's practice from primarily “transmission” (continuing the foundational stratum) and “absorption” (drawing from the influenced stratum) to an active, self-conscious stage of “creation” and “representation.” By composing and performing these works, the association aims to use Guangdong music—an “indigenous” art form—to tell “Macao's own” stories, to shape a distinctive and recognizable local sonic identity, and to make that identity an auditory manifesto of cultural belonging and a subjective narrative of local history.

These three historical strata coexist and interweave in present-day practice. Each performance thus becomes a dialogue with the “sound archives” of different historical periods, reactivating and reinterpreting their meanings in the present, and constituting a condensed, listenable history of local cultural encounters.

### 5.2 In-depth case study: *prosperous macao*—Hybrid grammar, bodily negotiation, and an open interpretive field

Composed by Liu Yinglin, a Macao Gaohu virtuoso and the association's chairman, reportedly around 2013 (also evidenced by a performance record marking Macao's 15th return anniversary in 2014), *Prosperous Macao* stands as the most ambitious cultural project among all the association's self-conscious efforts to shape a “Macao sound.” It transcends the category of a mere musical piece and becomes a comprehensive “cultural event” that integrates musical composition, bodily performance, meaning interpretation, and social reception.

1. Hybrid grammar of the musical text and historical metaphor. The work consciously experiments with cross-cultural musical language, and its “grammar” exhibits a profound hybridity. Its melodic core is firmly rooted in Guangdong music tradition, making extensive use of the highly expressive yifanxian mode and characteristic ornamentation (portamento, jiahua), ensuring an auditory “Cantonese base color.” However, its harmony and structure adopt Western harmonic systems and large-scale orchestral forces. This juxtaposition of “Chinese melodic skeleton, Western harmonic texture” is not a simple pastiche but creates a new language full of internal tension. When the plaintive, singing yifan melody of the gaohu floats above the rich Western harmonic progressions, the aesthetic experience is at once familiar and strange, intimate and alien. This sonic structure itself becomes a subtle aural metaphor for Macao's 400-year history of complex encounters—Eastern lyricism and Western rationality, individual destiny and grand collective narrative—colliding, dialoguing, and negotiating with each other in counterpoint.
2. Bodily negotiation in performance and the generation of a “New Habitus.” The hybridity of the musical score presents unprecedented bodily challenges for the performers, forcing them to coordinate two different musical mindsets and bodily habiti. The gaohu, dizi (bamboo flute), and other characteristic instruments must adjust their traditional sound production to “blend into” rather than “confront” the overall orchestral sound. At the same time, the performers must strive to maintain the flexible “internal pulse” (xinban) of Guangdong music within the strict rhythmic pulse of the Western-style orchestra. The conductor's rehearsal instructions are often “bilingual”—using orchestral terminology to demand balance between sections and, at the same time, using local metaphors (e.g., “like the distant sound of church bells”) to inspire the musicians. The repeated rehearsal process thus becomes a process of generating a new, composite bodily habitus, marking the point where cross-cultural fusion has penetrated the microscopic levels of bodily perception and motor programming.
3. Polyvalence of reception and interpretation: an open meaning field. The meaning of *Prosperous Macao* is co-constructed by multiple actors in its dissemination and reception, and its interpretation exhibits a rich polyvalence. Drawing on the researcher's interviews and field observations with diverse audience groups, different communities were found to interpret *Prosperous Macao* in significantly different ways. For the composer and the core members of the association, the work is regarded as an “epic of Macao sound.” For some seasoned traditional music enthusiasts, it is seen as challenging the stylistic boundaries of “traditional Guangdong music.” For younger citizens, it represents a sophisticated product of local culture. For outsiders, its hybrid quality reinforces the image of Macao as a “meeting point of East and West.” This openness and even contestation of interpretation precisely prove that *Prosperous Macao* is not a closed work with fixed meaning but an open field of ever-emerging signification. It continuously provokes multiple dialogues about Macao's cultural character, the boundaries of tradition, and contemporary identity. Its value lies precisely in its capacity to host and propel ongoing cultural negotiation.

This section, through analysis at the “context/historical integration” level, demonstrates how musical performance ethnography can integrate micro- and meso-level practices into a macro-level framework for comprehensive understanding. The historical stratification of the association's repertoire is itself a “listenable” history of local cultural encounters. A work such as *Prosperous Macao* marks a self-conscious shift from “transmitting in history” to “writing history with music”—an act of active, self-conscious identity construction and cultural representation. The Macao case shows that music has significant “textuality” and “agency” in identity construction. Local identity is not a static fact waiting to be “reflected” by music, but is continuously imagined, expressed, contested, and reaffirmed through creative musical practice. Music, with its capacity to transcend language, its direct emotional impact, and its complex symbolic coding, becomes a powerful medium for this meaning-making work.

## 6 Conclusion

This study, through an ethnographic examination of musical performance by the Macao Guangdong Music Friendship Association, reveals the practical logic by which a local musical genre survives and innovates in a cross-cultural urban setting. The findings show that the genre's vitality is first rooted in “embodied practice”: the stylistic essence is internalized as bodily habitus through hands-on discipline and daily rehearsal, and, under the long-term immersion in Macao's unique soundscape, a local aesthetic perception of “Macao taste”—characterized by composure and inclusiveness—is cultivated. At the same time, the association acts as an agentive cultural practitioner, strategically performing the roles of “culture guardian,” “cultural bridge,” and “cultural ambassador” in different social arenas—community festivals, educational settings, and international stages. This demonstrates that cultural identity is continuously negotiated and constructed within concrete social interactions and performance frames. Furthermore, the historical stratification of the association's repertoire and hybrid compositions such as *Prosperous Macao* indicate that music is not merely an echo of tradition but an agentive vehicle that participates in shaping local historical narratives and contemporary identity.

This study also represents a deep application of the ethnography of musical performance as a method. The constructed “Body–Event–Context” analytical framework effectively integrates micro-level embodied experience, meso-level social interaction, and macro-level historical context, providing an operational analytical path for understanding the “living” transmission and creative transformation of traditional music in urban settings. The case demonstrates that the contemporary vitality of tradition lies in practitioners maintaining its aesthetic core through embodied performance while actively reconstructing its meaning through strategic displays in different social fields. Cultural identity is embedded precisely in these everyday, embodied, and situated musical practices.

Future research may be extended along dimensions such as generational differences, media of transmission, and audience reception, so as to deepen our understanding of the contemporary transformation of traditional music.

## Data Availability

The original contributions presented in the study are included in the article/supplementary material, further inquiries can be directed to the corresponding author.

## Footnotes

1 Performance information for 2014, 2018, and 2024 mentioned in this chapter comes from oral accounts by association members as well as historical materials (photos, videos, concert programs) provided by the association. For the 2025 performances, unless otherwise noted, the July 13 event (80th anniversary of the Victory) and the December 14 event (26th anniversary of Macao's return) were directly observed by the author; other 2025 events were analyzed based on video recordings and member interviews.
